# Prediction models for postoperative cognitive dysfunction in adults: a systematic review of methodological quality and clinical applicability

**DOI:** 10.3389/fneur.2026.1786592

**Published:** 2026-06-17

**Authors:** Di Yang, Qian Li, Yunxia Zuo, Lei Yang

**Affiliations:** 1 Department of Anesthesiology, West China Hospital, Sichuan University, Chengdu, China; 2 Department of Anesthesiology, Sichuan Provincial People's Hospital, School of Medicine, University of Electronic Science and Technology of China, Chengdu, China

**Keywords:** critical appraisal, delayed neurocognitive recovery, postoperative neurocognitive disorder, prediction model, systematic review

## Abstract

**Background:**

Postoperative cognitive dysfunction (POCD) is associated with adverse outcomes. Although numerous prediction models have been developed, their methodological quality and clinical applicability remain uncertain.

**Methods:**

We systematically searched the MEDLINE, EMBASE, and Cochrane Central Register of Controlled Trials databases for studies that developed POCD prediction models in adults. Data extraction and quality assessment were performed using CHARMS (Checklist for Critical Appraisal and Data Extraction for Systematic Reviews of Prediction Modeling Studies) and PROBAST (Prediction model Risk Of Bias Assessment Tool).

**Results:**

From 2,060 initial records, 13 studies comprising 14 prediction models were included. Models were developed using logistic regression (*n* = 8) or machine learning (*n* = 6). Study sample size ranged from 82 to 687. Reported discriminatory performance was high [area under the receiver operating characteristic curve (AUC) range, 0.710–0.973]; however, these results were primarily derived from internally validated or unvalidated (rather than externally validated) models. The most common predictors of POCD were age (10 models) and preoperative hemoglobin concentration (four models). Twenty-three of the 30 predictors were measured preoperatively. The primary reasons for a high risk of bias were insufficient sample size and a lack of external validation. Only two models underwent temporal external validation, yielding diminished AUC (0.617 and 0.694). Furthermore, the incorporation of non-routine predictors such as functional magnetic resonance imaging and electroencephalography compromised the generalizability of the models to routine clinical settings, particularly because the routine use of these techniques would increase both preoperative waiting times and overall medical costs. Nonetheless, if sufficient predictive power is demonstrated, these non-routine predictors may be used more routinely in the clinic.

**Conclusion:**

Current prediction models for POCD show promising discriminatory performance, but their evaluation has been limited by short follow-up periods, a lack of external validation, and limited sample sizes, thus precluding their current clinical use. Future research should leverage large prospective cohorts, incorporate longitudinal multi-timepoint predictors that are routinely evaluated in clinical settings, extend follow-up to capture the full POCD course, and prioritize rigorous external validation to develop clinically applicable models.

**Systematic review registration:**

https://www.crd.york.ac.uk/PROSPERO/view/CRD42024622477

## 1 Introduction

Neurocognitive disorders after surgery are classified by the Perioperative Cognition Nomenclature Working Group into three categories: postoperative delirium, which occurs within 7 days of surgery; delayed neurocognitive recovery, which occurs within 30 days of surgery; and postoperative neurocognitive disorder, which extends from 30 days to 1 year after surgery (1, 2). Postoperative cognitive dysfunction (POCD) is an umbrella term that is commonly used to encompass both delayed neurocognitive recovery and postoperative neurocognitive disorder. It describes a decline in cognitive functions, such as memory, attention, and executive function, that occurs within 1 year post-surgery (3). Although POCD is not yet formally defined and there is no consensus for its diagnosis or testing, it is a commonly used research construct (4). Despite POCD no longer being the recommended diagnostic term within the updated perioperative neurocognitive disorder nomenclature, we used this term in the present review because it is still widely used in research as a legacy construct, particularly in studies published before (or shortly after) the nomenclature change.

The pathogenesis of POCD remains relatively unclear, although it seems that the disorder arises from a combination of factors such as age, type of surgery, mode and duration of anesthesia, and pain intensity (5–7). Inflammation (8–10), neuronal apoptosis, and impaired synaptic plasticity are also considered to be involved in POCD development (7), and POCD is reportedly associated with pre-surgical memory impairment (11). The prevalence of POCD has recently been reported as 23% at postoperative day 7, 16% at 1 month, 10% at 3 months, and 3% at 1 year (12). POCD is associated with an increased incidence of complications, prolonged hospital stay, and reduced quality of life (1). It also elevates the risk of persistent cognitive impairment, which shares pathological mechanisms with Alzheimer's disease (7). Importantly, the accurate perioperative identification of patients at high risk of experiencing POCD and the early implementation of appropriate interventions are crucial for improving patient outcomes and reducing healthcare burden.

Given the multifactorial etiology of POCD—involving patient-, surgery-, anesthesia-, and inflammation-related factors—as well as the availability of perioperative clinical, neurophysiological, and neuroimaging data, POCD represents a suitable target for predictive modeling and early perioperative risk stratification. Recent advances in data analytics have enabled POCD prediction models to incorporate not only demographic and clinical variables (13, 14) but also neuroimaging and EEG data (15, 16), which can reflect neuroinflammation as well as related changes in brain function (17). However, methodological challenges such as heterogeneity in POCD definitions, variability in neuropsychological testing protocols, inconsistent follow-up intervals, and the frequent absence of external validation mean that the methodological quality and clinical applicability of these models remain uncertain. We therefore conducted a systematic review and critical appraisal of studies that have developed POCD prediction models.

## 2 Methods

This study was registered with PROSPERO (CRD42024622477). The present review followed the registered PROSPERO protocol without deviations and was reported in line with the Preferred Reporting Items for Systematic reviews and Meta-Analyses (PRISMA) 2020 guidelines (18).

### 2.1 Literature search strategy

We conducted systematic searches via MEDLINE/Ovid (publication date: 1946 to 14 May 2025), EMBASE/Ovid (publication date: 1974 to 14 May 2025), and Cochrane Central Register of Controlled Trials/Ovid (publication date: on or before April 2025). Search terms included “postoperative cognitive complications,” “delayed neurocognitive recovery,” “postoperative cognitive dysfunction,” “postoperative/post-operative/postsurgery cognitive decline/impairment,” “statistical model,” “nomogram,” “linear models,” “clinical decision rule,” and “machine learning.” Only articles written in English were included because English is the most widely used language in the current biomedical research field, and the core literature of most high-impact journals and major databases (e.g., PubMed, Web of Science, EMBASE) is published in English. Detailed search strategies are provided in Appendix 1.

### 2.2 Inclusion and exclusion criteria

On the basis of the PICO framework, studies that met the following criteria were included. Patients: Adult patients undergoing any surgical procedure. Intervention: Development of any POCD prediction model. Comparison: Not applicable. Outcome: The predicted outcome was defined as cognitive decline, assessed using neuropsychological scales, occurring between 7 days and 1 year after surgery. Study: No restrictions were applied regarding study design.

Studies that evaluated the predictive value of single factors (e.g., the association of age or a single biomarker with POCD) were excluded because such studies are essentially correlational analyses and do not constitute true prediction models. Reviews, systematic reviews, and preprint literature were also excluded. This strategy was used to ensure that the present systematic review was focused on tools that can be used for clinical individual risk stratification.

### 2.3 Study screening and selection

We used EndNote X9 for reference management and performed automatic deduplication using this software. Two independent reviewers (DY and QL) then screened the titles and abstracts of the retrieved records. If the eligibility of a study could not be determined based on the title and abstract, the full-text article was obtained and thoroughly assessed. The full texts of all potentially relevant studies were then thoroughly assessed independently by both reviewers to make a final decision regarding inclusion. Any disagreements during the screening process were resolved through discussion. If consensus could not be reached, a third independent reviewer (LY) was consulted to make the final determination.

### 2.4 Data extraction and quality assessment

A structured electronic data extraction form, adapted from a previously published template (19), was used to ensure consistent and comprehensive data collection. First, pilot data extraction was performed on three articles. Next, the two reviewers independently extracted data from each included study using CHARMS (Checklist for Critical Appraisal and Data Extraction for Systematic Reviews of Prediction Modeling Studies) (20) before comparing the results and making any necessary corrections or revisions. This process captured essential details regarding model development (e.g., sample size, handling of missing data, and model type), predictor variables (definitions and measurement methods), and model performance metrics (e.g., discrimination, calibration, and overall accuracy). If information was missing or unclear, the reviewers first consulted the original articles and supplementary materials, contacting the authors when necessary. If the information remained missing, it was marked as “not reported” without subjective imputation. Any discrepancies between the two reviewers were resolved through consensus or by consulting a third reviewer (when necessary).

The risk of bias and concerns regarding the applicability of each prediction model were rigorously evaluated using PROBAST (Prediction model Risk Of Bias Assessment Tool) (21). PROBAST examines four key domains: participants, predictors, outcome, and analysis. Each model was independently judged by the two reviewers as having “low,” “high,” or “unclear” risk of bias, with any discrepancies resolved through consensus or by consulting a third reviewer. Similarly, the applicability of the model to the review question was assessed.

### 2.5 Descriptive analyses

There was substantial clinical and methodological heterogeneity among the included studies. For example, different models incorporated different types of predictors, and the time points for postoperative cognitive function assessment varied across studies, leading to variations in the included variables, meaning that a meta-analysis was not feasible. We therefore performed a descriptive synthesis of all findings and summarized the following key aspects: population characteristics, sample size, predictors, statistical methods used for model development and validation, and results of the methodological quality assessment using PROBAST, which evaluated both the risk of bias and concerns regarding applicability for each prediction model.

## 3 Results

**Figure 1:**
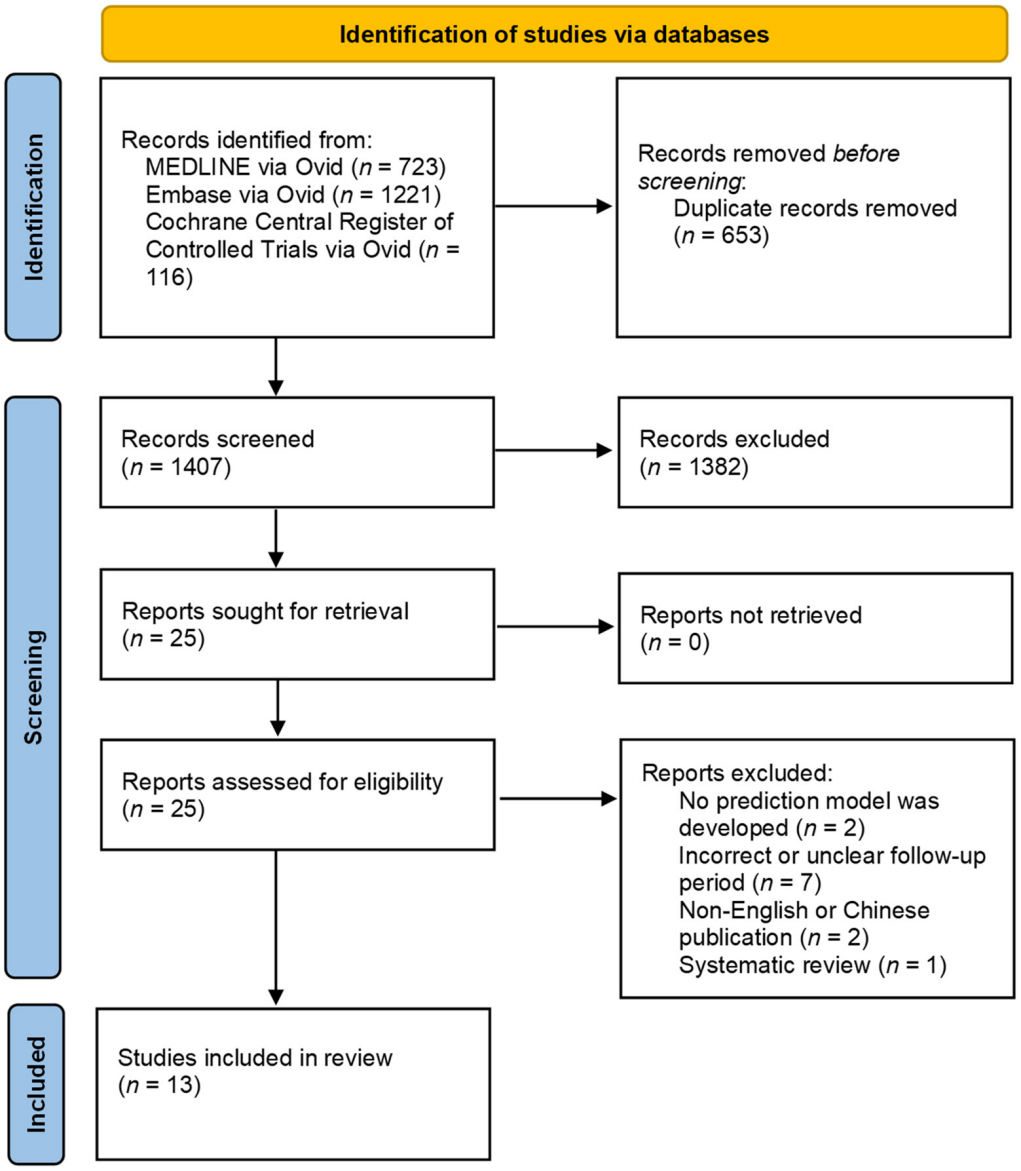
Flow diagram of study screening.

Through a systematic search, 2,060 records were identified. After removing duplicates, 1,407 unique records remained. Through title and abstract screening, 25 full-text articles were assessed for eligibility. Following a detailed full-text review, 13 studies that reported the development of 14 distinct prediction models were included (Figure 1) (13–16, 22–30). Of these 13 studies, 11 were conducted in China. One study described both a model developed using preoperative variables alone, and a model that incorporated both preoperative and postoperative variables (28). Because of considerable heterogeneity among the included studies with respect to demographics, physical conditions, laboratory values, and imaging metrics (for example, Table 1 shows wide variation in study design, surgery type, age, predicted outcome, outcome measurements, and follow-up periods), a quantitative synthesis was not feasible. Instead, a descriptive synthesis was conducted to summarize the findings.

**Table 1:** Characteristics of included studies.

References	Study design	Enrollment period	Study region	Surgery type	Age (years)	Predicted outcome	Outcome measurements	Follow-up period
Zhang et al. (25)	Prospective cohort	February 2021–March 2022	China	Non-cardiac surgery	≥650	DNR	Neuropsychological tests	Postoperative 7 days
Wang et al. (14)	Retrospective cohort	March 2020–July 2022 (modeling group), January 2023–July 2023 (validation group)	China	Laparoscopy for nodular bowel cancer	≥500	DNR	MMSE	Postoperative 7 days
Wang et al. (13)	Retrospective cohort	January 2014–January 2020	China	Radical gastric cancer surgery	70–930	Postoperative cognitive dysfunction	MMSE	Postoperative 7 days
Verdonk et al. (23)	Randomized trial	20 March 2017–28 May 2019	France	Major orthopedic surgery	≥60 0	Postoperative cognitive decline	Cognitive tests	Postoperative 7 days
Luo et al. (26)	Retrospective cohort	March 2018–July 2021	China	Laparoscopic colorectal cancer surgery	≥500	DNR	MMSE	Postoperative 7 days
Jiang et al. (30)	Nested case–control	September 2017–February 2019	China	Non-cardiac surgery	≥60	DNR	Neurocognitive tests	Postoperative 7–14 days
Jiang et al. (24)	Nested case–control	September 2017–February 2019	China	Non-cardiac surgery	≥60	DNR	Neurocognitive tests	Postoperative 7–14 days
Huang et al. (27)	Case–control	March 2018–October 2021	China	Gastrointestinal tumor resection	>60	POCD	MMSE	Postoperative 7 days
Wu et al. (15)	Nested case–control	September 2017–February 2019	China	Non-cardiac surgery	≥600	DNR	Neurocognitive tests	Postoperative 7–14 days
Li et al. (28)	Retrospective cohort	May 2020–May 2021	China	Non-cardiac surgery	≥600	POCD	MMSE	Postoperative 6 months
Geraedts et al. (16)	Prospective cohort	May 2017–July 2019	Netherlands	Bilateral subthalamic nucleus deep brain stimulation	>180	Postoperative cognitive deterioration	Cognitive tests	Postoperative 1 year
Yong et al. (29)	Prospective cohort	May 2018–May 2022	China	Laparoscopic radical gastrectomy	≥65	DNR	Cognitive tests	Postoperative 30 days
Xie et al. (22)	Retrospective cohort	May 2020–May 2021	China	Non-cardiac surgery	≥60	POCD	MMSE	Postoperative 3 months

DNR, delayed neurocognitive recovery; MMSE, mini-mental state examination; POCD, postoperative cognitive dysfunction.

Among the included studies, four used a prospective cohort design (16, 23, 25, 29), five were retrospective (13, 14, 22, 26, 28), and four adopted a nested case–control or case–control methodology (15, 24, 27, 30). All studies exclusively involved patients undergoing non-cardiac surgery and were conducted within a single center. Only one study included participants under the age of 50 years (16). Regarding the timing of outcome assessments, nine studies evaluated cognitive outcomes within 7–14 days after surgery (13–15, 23–27, 30); the other four used postoperative endpoints of 1 month (29), 3 months (22), 6 months (28), and 1 year (16). Detailed characteristics of the included studies are presented in Table 1.

Of the included prediction models, eight were developed using logistic regression methodology (13, 14, 25–29), whereas six adopted a machine learning approach (15, 16, 22–24, 30). The largest sample size among the individual studies was 687 (13). The discriminatory performance of the models, as measured by the area under the receiver operating characteristic curve (AUC), ranged from 0.710 to 0.973 across all models. Calibration metrics were not reported in five studies: one study using serum immunological markers as predictors (23), three studies using functional magnetic resonance imaging (fMRI) (15, 24, 30), and one study based on EEG data (16). Apart from one study (13), all reported some form of internal validation. Notably, two studies that incorporated predictors related to demographics, comorbidities, and surgery/anesthesia factors performed temporal external validation, yielding reduced AUC values of 0.617 and 0.694 (14, 26). Detailed characteristics of the prediction models are summarized in Table 2.

**Table 2:** Characteristics of the prediction models.

References	Modeling method	Events/sample size (%)	No. predictors		EPV or EPP	Selection of candidate predictors	Selection of final predictors	Type of validation	Performance measures	Type of predictors	Timing of predictor measurement
			**Cand**.	**Final**							
Zhang et al. (25)	Logistic regression	33/138 (23.9)	34	9	1.0	Based on prior knowledge and univariable associations	Enter method	Int: Bootstrap and split-sample validation Ext: None	Calibration: Calibration plot/Hosmer–Lemeshow test Discrimination: AUC 0.801 (95% CI: 0.683–0.918) Overall: Not evaluated	Demographic (age, education), surgery/anesthesia-related (VAS, gastrointestinal), cognitive function (HVLT^*^2, DDST, JLOT, cognitive frailty) variables	Preoperative and postoperative
Wang et al. (14)	Logistic regression	35/227 (15.4)	8	4	4.4	Unclear	Unclear	Int: Bootstrap Ext: Temporal	Calibration: Calibration plot/Hosmer–Lemeshow test Discrimination: AUC 0.757 (95% CI: 0.676–0.839) Overall: Not evaluated	Demographic (age, education), comorbidities (diabetes), and surgery/anesthesia-related (rScO_2_)	Preoperative and intraoperative
Wang et al. (13)	Logistic regression	141/687 (20.5)	15	7	9.4	Unclear	Unclear	Int: No information Ext: No information	Calibration: Hosmer–Lemeshow test Discrimination: AUC 0.820 (95% CI: 0.742–0.899) Overall: Not evaluated	Overall physical condition (ASA, PG-SGA), surgery/anesthesia-related (dexmedetomidine, BIS, operation time), demographic (age), and lab (Hb) values	Preoperative and intraoperative
Verdonk et al. (23)	Machine learning techniques	11/26 (42.3)	1485	11	0.0	All available predictors	LASSO selection	Int: Cross-validation Ext: None	Calibration: Not evaluated Discrimination: AUC 0.80 (0.54–0.9) Overall: Not evaluated	Immunological data	Preoperative
Luo et al. (26)	Logistic regression	31/204 (15.2)	7	4	4.4	Unclear	Unclear	Int: Bootstrap Ext: Temporal	Calibration: Calibration plot/Hosmer–Lemeshow test Discrimination: AUC 0.751 (95% CI, 0.661–0.842) Overall: Not evaluated	Demographic (age, education), comorbidities (diabetes), and surgery/anesthesia-related (lrSO_2_V)	Preoperative and intraoperative
Jiang et al. (30)	Machine learning techniques	16/74 (21.6)	11	5	2.0	Based on prior knowledge	Embedded feature selection	Int: Cross-validation and split-sample validation Ext: None	Calibration: Not evaluated Discrimination: AUC 0.958 Overall: Not evaluated	Neuroimaging (fMRI)	Preoperative
Jiang et al. (24)	Machine learning techniques	16/74 (21.6)	18	Unclear	0.9	Based on prior knowledge	Embedded feature selection	Int: Cross-validation and split-sample validation Ext: None	Calibration: Not evaluated Discrimination: AUC 0.864 Overall: Not evaluated	Neuroimaging (fMRI)	Preoperative
Huang et al. (27)	Logistic regression	79/369 (21.4)	8	7	9.9	Based on prior knowledge and univariable associations	Stepwise selection	Int: Bootstrap Ext: None	Calibration: Calibration plot/Hosmer–Lemeshow test Discrimination: AUC 0.710 (95% CI = 0.645–0.775) Overall: Not evaluated Clinical utility: Decision curve analysis	Demographic (age, BMI), lab values (WBC, Hb), comorbidities (cerebrovascular disease), and surgery/anesthesia-related (blood loss, operation time)	Preoperative and intraoperative
Wu et al. (15)	Machine learning techniques	16/74 (21.6)	390	40	0.0	All available predictors	Sparse representation	Int: Cross-validation and split-sample validation Ext: None	Calibration: Not evaluated Discrimination: AUC 0.956 Overall: Not evaluated	Neuroimaging (fMRI)	Preoperative
Li et al. (28)	Logistic regression	23/415 (5.5)	12	4	1.9	Based on univariable associations	Stepwise selection	Int: Cross-validation Ext: None	Calibration: Calibration plot/Hosmer–Lemeshow test Discrimination: AUC 0.947 (95% CI 0.913–0.980) Overall: Not evaluated Clinical utility: Decision curve analysis	Demographic (age), comorbidities (arrhythmia, diabetes), and lab values (Hb)	Preoperative
Geraedts et al. (16)	Logistic regression	23/415 (5.5)	25	4	0.9	Based on univariable associations	Stepwise selection	Int: Cross-validation Ext: None	Calibration: Calibration plot/Hosmer–Lemeshow test Discrimination: AUC 0.973 (95% CI 0.949–0.996) Overall: Not evaluated Clinical utility: Decision curve analysis	Demographic (age), comorbidities (arrhythmia), lab values (Hb), and surgery/anesthesia-related (VAS)	Preoperative and postoperative
Yong et al. (29)	Machine learning techniques	25/60 (41.6)	16674	18	0.0	All available predictors	Boruta selection	Int: Cross-validation and split-sample validation Ext: None	Calibration: unclear Discrimination: ROC curve Overall: Not evaluated Clinical utility: Not evaluated	EEG	Preoperative
Xie et al. (22)	Logistic regression	73/312 (23.4)	9	5	8.1	Based on univariable associations	Unclear	Int: Split-sample validation Ext: None	Calibration: Calibration plot Discrimination: AUC 0.863 Overall: Not evaluated Clinical utility: Decision curve analysis	Demographic (age), physical condition (NRS2002), and lab values (NLR, AFR, and PNI)	Preoperative
Zhang et al. (25)	Machine learning techniques	35/415 (8.4)	26	6	0.9	Based on prior knowledge and univariable associations	LASSO	Int: Cross-validation and split-sample validation Ext: None	Calibration: Calibration plot Discrimination: AUC 0.950 (95% CI: 0.875–1.000) Overall: Brier score 0.038 Clinical utility: Not evaluated	Demographic (age), lab values (Hb), surgery/anesthesia-related (intraoperative hypotension, blood loss, VAS, surgery duration)	Preoperative, intraoperative, and postoperative

AFR, albumin-to-fibrinogen ratio; ASA, American Society of Anesthesiologists; AUC, area under the curve; BIS, bispectral index; BMI, body mass index; CI, confidence interval; DDST, digits symbol substitution test; EEG, electroencephalogram; EPP, events per predictor; EPV, events per variable; Ext, external; fMRI, functional magnetic resonance imaging; Hb, hemoglobin; HVLT, Hopkins verbal learning test; Int, internal; JLOT, judgment of line orientation test; LASSO, least absolute shrinkage and selection operator; lrScO_2_v, maximum variability of cerebral saturation oxygenation during left cerebral operation; NLR, neutrophil-to-lymphocyte ratio; NRS, nutritional risk screening; PG-SGA, patient-generated subjective global assessment; PNI, prognostic nutritional index; ROC, receiver operating characteristic; rScO_2_, regional cerebral saturation oxygenation; VAS, visual analog scale; WBC, white blood cell count.

**Figure 2:**
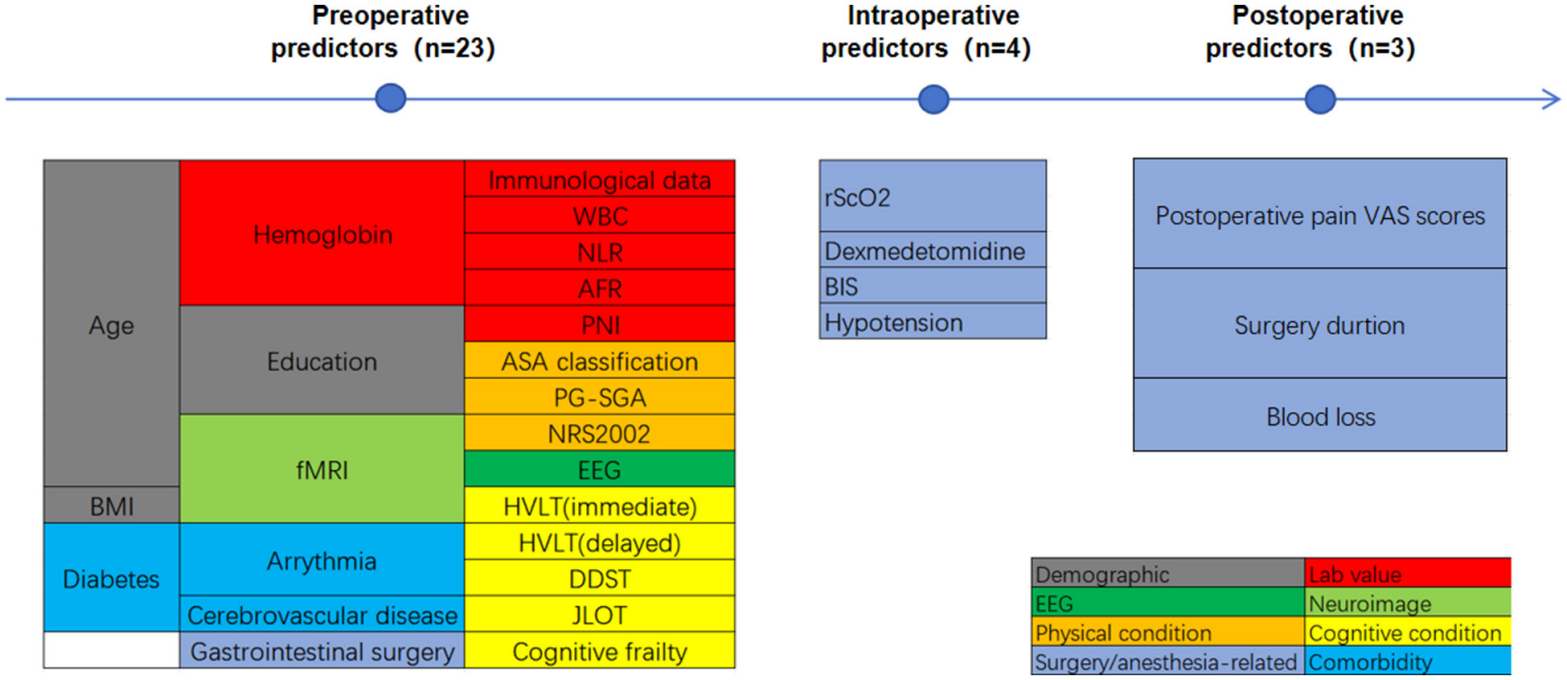
Summary of the predictors used in the included prediction models (13–15, 22–30). The height of each column represents the frequency of the occurrence of the predictor. AFR, albumin-to-fibrinogen ratio; ASA, American Society of Anesthesiologists; BIS, bispectral index; BMI, body mass index; DDST, digits symbol substitution test; EEG, electroencephalogram; fMRI, functional magnetic resonance imaging; HVLT, Hopkins verbal learning test; JLOT, judgment of line orientation test; lrScO2v, maximum variability of cerebral saturation oxygenation during left cerebral operation; NLR, neutrophil-to-lymphocyte ratio; NRS, nutritional risk screening; PG-SGA, patient-generated subjective global assessment; PNI, prognostic nutritional index; rScO2, regional cerebral saturation oxygenation; VAS, visual analog scale; WBC, white blood cell count.

The predictors that were incorporated into the prediction models covered a broad spectrum of variables, including demographic factors, comorbidities, surgery/anesthesia-related factors, cognitive status, physical function, neuroimaging, EEG data, and laboratory values. Age and preoperative hemoglobin concentration were the most frequently occurring predictors, appearing in 10 and 4 models, respectively. For these predictors, a higher age and lower preoperative hemoglobin levels were associated with a greater risk of POCD. Education level, the presence of diabetes, fMRI findings, postoperative visual analog scale score for pain, and surgery duration each appeared in three models. In terms of timing, most predictors (23 variables) were obtained before surgery, four predictors were acquired during surgery, and three after. Most predictors were relatively routine clinical measures (for example, demographic variables and comorbidities are usually readily available in clinical practice, and surgery/anesthesia-related variables and lab values are relatively easy to obtain); however, neuroimaging and EEG require specialized testing before surgery and are not routinely performed. A comprehensive summary of all predictors is provided in Figure 2.

The quality assessment results are presented in Table 3. The primary reasons for a high risk of bias were an insufficient follow-up period, limited sample size, and lack of external validation. Furthermore, immunological tests, EEG, and fMRI are not routinely performed in clinical practice, therefore the incorporation of these predictors substantially limits the overall applicability of the models in real-world settings.

**Table 3:** PROBAST (prediction model risk of bias assessment tool) results of included studies.

References	Risk of bias				Applicability			Overall	
1. Participants	2. Predictors	3. Outcome	4. Analysis	1. Participants	2. Predictors	3. Outcome	Risk of bias	Applicability
Zhang et al. (25)	+	+	–	–	+	+	+	–	+
Wang et al. (14)	+	+	–	–	–	+	+	–	–
Wang et al. (13)	+	+	–	–	–	+	+	–	–
Verdonk et al. (23)	+	+	–	–	–	+	+	–	–
Luo et al. (26)	+	+	–	+	–	+	+	–	–
Jiang et al. (30)	+	+	–	+	+	–	+	–	–
Jiang et al. (24)	+	+	–	+	+	–	+	–	–
Huang et al. (27)	+	?	–	?	–	+	+	–	–
Wu et al. (15)	+	+	–	–	+	–	+	–	–
Li et al. (28)	+	?	–	–	+	+	+	–	+
Geraedts et al. (16)	+	+	?	–	–	+	+	–	–
Yong et al. (29)	+	+	–	–	–	+	+	–	–
Xie et al. (22)	?	?	–	–	+	+	?	–	?

Green (+) represents low risk, red (-) represents high risk, and yellow (?) represents unclear risk.

## 4 Discussion

This systematic review included 13 studies that reported the development of 14 prediction models using either logistic regression or machine learning. A notable limitation was the absence of robust external validation across all studies. Owing to the considerable diversity in predictor variables, a descriptive synthesis was performed rather than a quantitative analysis. Nevertheless, several important findings can be summarized.

First, although some of the included studies aimed to predict delayed neurocognitive recovery, cognitive assessment was only conducted between 7 and 14 days after surgery. This short-term follow-up period is likely insufficient to capture the full trajectory of postoperative neurocognitive decline and may increase overlap with postoperative delirium, thereby raising concerns regarding outcome misclassification. Consequently, some models may preferentially predict transient acute postoperative cognitive disturbances rather than persistent cognitive impairment, potentially reducing the validity, clinical interpretability, and generalizability of the reported prediction models. Moreover, all studies relied on a single postoperative time point for outcome assessment. Given that cognitive function often fluctuates over time (4), a one-time, cross-sectional evaluation may fail to accurately reflect the dynamic trajectory of postoperative cognitive changes, and therefore provides an incomplete clinical picture. Reliance on a single postoperative assessment time point limits the ability of prediction models to capture dynamic cognitive trajectories over time. Patients may exhibit heterogeneous patterns of recovery or deterioration, meaning that single time-point assessments may underestimate risk in individuals with progressive decline while overestimating risk in those with transient impairment followed by recovery. Consequently, such models may provide an incomplete representation of postoperative neurocognitive outcomes and reduce the clinical utility of individualized risk prediction. The findings of that previous study support the use of cognitive trajectory as a feasible and meaningful outcome measure (31). Future studies should therefore adopt longitudinal cognitive monitoring over extended periods to enhance the accuracy and clinical relevance of outcome assessment. A major obstacle to long-term cognitive monitoring is the reliance on conventional tools such as the Mini-Mental State Examination (32) and the Montreal Cognitive Assessment (33), which require face-to-face interviews and may increase attrition rates. Fortunately, simplified instruments are emerging, such as the AD8 informant interview (34), suitable for the early screening of mild cognitive impairment, and the telephone Montreal Cognitive Assessment (35), which enables remote evaluation. The implementation of these streamlined tools may significantly improve the feasibility and sustainability of long-term cognitive monitoring after surgery.

Second, it is noteworthy that the identified pool of candidate predictors was heavily skewed toward the preoperative period. Twenty-three variables were identified preoperatively, whereas only seven were collected during or after surgery. This imbalance may be partly attributed to the observational nature of most existing studies, in which preoperative data are more readily accessible. Similarly, a 2023 meta-analysis of the perioperative risk factors associated with POCD identified that 74% of reported risk factors were preoperative measures (36). However, given that the development of POCD is a dynamic process that occurs throughout the perioperative period, if certain preoperative risk factors (such as specific comorbidities or abnormal laboratory results) emerge or worsen after surgery, their inclusion may also enhance predictive accuracy. Therefore, future research should prioritize the development of dynamic, multi-dimensional databases that systematically capture longitudinal data across all perioperative phases. Additionally, statistical methods such as “landmark analysis” and “joint models” can be used to identify variables at multiple time points (37, 38). Such approaches are expected to improve the accuracy of POCD prediction.

Third, in addition to routinely available clinical data such as demographics and comorbidities, non-routine factors including immunological markers, EEG, and fMRI were also demonstrated to have predictive value. From a mechanistic perspective, the neuroinflammatory response is recognized as a key pathophysiological factor in POCD (39, 40), and alterations in brain function may represent its neurobiological substrate (17). Therefore, incorporating these biomarkers into prediction models appears physiologically justified. However, from a clinical applicability standpoint, the inclusion of such non-routine predictors would inevitably increase healthcare costs and operational complexity. Therefore, in addition to conducting rigorous external validation, future studies should consider developing a stratified prediction framework. Such an approach may help to balance predictive accuracy with practicality; for example, by reserving advanced biomarkers for high-risk populations or complex clinical scenarios.

Fourth, the methodological limitations observed in the included studies—particularly, insufficient sample size and a lack of external validation—substantially restrict the clinical generalizability of the prediction models. Although the included studies used either logistic regression or machine learning to develop models with reportedly high predictive performance, temporal validation was performed in only two studies; the other 11 studies lacked any form of external validation. When evaluating model performance, external validation (i.e., assessing the predictive performance of a model in a relevant dataset that was not used in the development process) is very important for clinical translation (41). The absence of external validation and the relatively small sample sizes across these studies limit the reliability and applicability of the models (42). In recent years, the establishment of several large-scale study cohorts (43, 44) offers a promising opportunity to address these methodological shortcomings. Future models developed within such frameworks may overcome current limitations and enhance the robustness and clinical utility of POCD prediction. Notably, when considering the clinical translation of POCD prediction models, a recently published step-by-step guide for developing clinical prediction models emphasizes that studies should clearly define the aim and users, select appropriate data sources, address missing data, explore alternative modeling options, and assess model performance (45).

Fifth, the finding that age, preoperative hemoglobin concentration, diabetes, and education were the most commonly used non-surgery/anesthesia-related predictors in the included models may have clinical implications. Similarly, a recently published retrospective database study noted age and biochemical tests as the top two risk factors associated with POCD, and diabetes was also included in the top 20 (46). Aging is the only known risk factor for delayed neurocognitive recovery (25), and older age is associated with a greater risk of developing POCD (47). Moreover, a recent systematic review and meta-analysis revealed that patients with diabetes have a significantly higher POCD risk compared with those without diabetes, which may be because diabetes is associated with inflammation (48). Lower preoperative hemoglobin is also reportedly associated with POCD (49, 50), possibly because lower hemoglobin levels may result in brain hypoxia (51). Therefore, future models should consider including age, preoperative hemoglobin concentration, diabetes, and education as potential predictors of POCD.

Sixth, we note that 11 of the 13 included studies were conducted in China. This is likely because China is a lead producer of research into POCD. A recent bibliometric analysis of basic POCD research in the last decade revealed that only two countries had published more than 10 studies: the USA, with 56 studies, and China, with 444 studies (52). However, given that the majority of included studies came from China, the results of our analysis may not be generalizable to other regions. For example, the studies conducted in China may differ from those in other regions in terms of genetic factors of participants, perioperative management, and the cultural adaptability of cognitive assessment tools. The extrapolation of these models to other populations therefore requires caution. Nonetheless, we note that the methodological issues and recommendations that were identified are not influenced by geographic factors. Together, these findings suggest that more research into POCD prediction should be performed in other geographical settings to ensure that results are applicable worldwide.

Seventh, five included studies did not report calibration metrics. A lack of calibration assessment means that even if a model has good discrimination, there may be systematic bias between the predicted probabilities and actual risks. Importantly, this greatly reduces confidence in the absolute predictive accuracy of a model and limits its clinical applicability.

The present review has several limitations. First, substantial heterogeneity was observed among the included models regarding predictor selection, cognitive assessment methods, and follow-up duration, which precluded a quantitative synthesis. Second, the limited external validation and small sample sizes of the included prediction models mean that the results of the included studies are not robust. Small sample sizes may lead to overfitting, and studies should include at least 10 events per variable and use internal validation methods (e.g., cross-validation or bootstrapping) to assess the degree of overfitting. Moreover, model development should be accompanied by at least one external validation result to be recommended for clinical use. Third, the reliance on non-routine predictors (e.g., fMRI or EEG) in many studies limits the clinical applicability of any findings. Unless such predictors can significantly improve model prediction accuracy, priority should be given to developing models that include routine variables. Fourth, the relatively short follow-up periods of the included studies mean that POCD was likely missed in some patients (e.g., those who developed cognitive impairments later in the postoperative period). Fifth, because only English-language publications were included, the findings may be subject to language bias and have limited generalizability. Sixth, the focus on POCD—a relatively ambiguous term that is no longer commonly used—may limit the value of any analyses. Seventh, the lack of predictors that reflect dynamic change combined with a focus on preoperative data (with little emphasis on factors such as surgical invasiveness and postoperative complications) means that the included models may be missing important data for predicting POCD.

## 5 Conclusions

We identified age and preoperative hemoglobin concentration as the most frequently used predictors in models for POCD. However, we noted many methodological limitations in the relevant studies, such as an insufficient follow-up period, limited sample size, and lack of external validation, which substantially limit our confidence in these predictors. Structural barriers in POCD prediction research also exist, such as inconsistent definitions, non-standardized assessments, and variable outcome criteria and timing. These issues prevent the included prediction models from meeting clinical decision-making requirements because the reliability of the results cannot be ascertained. Moreover, some studies used only brief screening scales (e.g., the Mini-Mental State Examination), whereas others used comprehensive neuropsychological test batteries covering multiple cognitive domains; these inconsistencies limit the clinical applicability of the models. The present review therefore presents a clear path forward for the development of models to predict POCD. Specifically, future research should leverage longitudinal data from large prospective cohort studies; extend follow-up to cover the full disease course of POCD; integrate routinely clinically evaluated predictors from preoperative, intraoperative, and postoperative phases to develop dynamic prediction models; and perform rigorous external validation to facilitate the clinical translation of reliable predictive models. In particular, longitudinal cognitive follow-up, dynamic perioperative data collection, and rigorous external validation are essential prerequisites for the development of clinically usable models. When such prediction models have been validated in multicenter settings, they should be integrated into clinical decision support systems to enhance early prediction and facilitate treatment of POCD. Nonetheless, we must acknowledge that such integration will only be feasible once methodological rigor and generalizability have been demonstrated.

## Data Availability

The original contributions presented in the study are included in the article/Supplementary material, further inquiries can be directed to the corresponding author.
