# Supplementary material for: Prediction models for postoperative cognitive dysfunction in adults: a systematic review of methodological quality and clinical applicability
Source: Front Neurol. 2026 Jun 17;17:1786592. doi: 10.3389/fneur.2026.1786592 (PMC13318596; doi:10.3389/fneur.2026.1786592)
Supplement: Supplementary file 1 [file Supplementary_file_1.docx]

Ovid MEDLINE(R) ALL <1946 to May 14, 2025>

1 exp Postoperative Cognitive Complications/ or Postoperative Cognitive Complications.mp. or delayed neurocognitive recovery.mp. or postoperative cognitive dysfunction.mp. or ((Postoperative or post-operative or postsurgery) and (cognitive decline or cognitive impairment)).mp.

2 statistical model.mp. or exp Models, Statistical/ or nomogram.mp. or exp Nomograms/ or Linear Models.mp. or exp linear models/ or clinical decision rule.mp. or exp Clinical Decision Rules/ or machine learning.mp. or exp Machine Learning/ or predict$.mp.

3 1 and 2

4 limit 3 to animals

5 3 not 4

Embase <1974 to 2025 May 14>

1 exp Postoperative Cognitive Complications/ or Postoperative Cognitive Complications.mp. or delayed neurocognitive recovery.mp. or postoperative cognitive dysfunction.mp. or ((Postoperative or post-operative or postsurgery) and (cognitive decline or cognitive impairment)).mp.

2 statistical model.mp. or exp Models, Statistical/ or nomogram.mp. or exp Nomograms/ or Linear Models.mp. or exp linear models/ or clinical decision rule.mp. or exp Clinical Decision Rules/ or machine learning.mp. or exp Machine Learning/ or predict$.mp.

3 1 and 2

4 limit 3 to animals

5 3 not 4

EBM Reviews - Cochrane Central Register of Controlled Trials <April 2025>

1 exp Postoperative Cognitive Complications/ or Postoperative Cognitive Complications.mp. or delayed neurocognitive recovery.mp. or postoperative cognitive dysfunction.mp. or ((Postoperative or post-operative or postsurgery) and (cognitive decline or cognitive impairment)).mp.

2 statistical model.mp. or exp Models, Statistical/ or nomogram.mp. or exp Nomograms/ or Linear Models.mp. or exp linear models/ or clinical decision rule.mp. or exp Clinical Decision Rules/ or machine learning.mp. or exp Machine Learning/ or predict$.mp.

3 1 and 2
